# The multidimensional legal nature of personal genomic sequence data: A South African perspective

**DOI:** 10.3389/fgene.2022.997595

**Published:** 2022-11-09

**Authors:** Donrich W. Thaldar, Beverley A. Townsend, Dusty-Lee Donnelly, Marietjie Botes, Amy Gooden, Joanne van Harmelen, Bonginkosi Shozi

**Affiliations:** ^1^ School of Law, University of KwaZulu-Natal, Durban, South Africa; ^2^ York Law School, University of York, York, United Kingdom; ^3^ SnT Interdisciplinary Centre for Security, Reliability Security and Trust, University of Luxembourg, Luxembourg, Luxembourg; ^4^ ENS Africa, Cape Town, South Africa; ^5^ School of Law, Institute for Practical Ethics, University of California, San Diego, San Diego, CA, United States

**Keywords:** data, human genome, intellectual property, ownership, privacy, property

## Abstract

This article provides a comprehensive analysis of the various dimensions in South African law applicable to personal genomic sequence data. This analysis includes property rights, personality rights, and intellectual property rights. Importantly, the under-investigated question of whether personal genomic sequence data are capable of being owned is investigated and answered affirmatively. In addition to being susceptible of ownership, personal genomic sequence data are also the object of data subjects’ personality rights, and can also be the object of intellectual property rights: whether on their own *qua* trade secret or as part of a patented invention or copyrighted dataset. It is shown that personality rights constrain ownership rights, while the exploitation of intellectual property rights is constrained by both personality rights and ownership rights. All of these rights applicable to personal genomic sequence data should be acknowledged and harmonized for such data to be used effectively.

## 1 Introduction

Debates on the governance of human genomic data are often cast in a one-dimensional mold with respect to the legal nature of the subject matter. Human genomic data are conceived of in the data’s property rights dimension (which includes concepts such as data ownership and custodianship), or in its personality rights dimension (which includes privacy rights), or in its intellectual property law dimension (which includes patentable inventions, copyright in datasets, and trade secrets). Sometimes, two of these dimensions—academic subfields within law—are considered together. But all three of these dimensions and the interplay between them are rarely, if ever, considered together. The purpose of this article is to provide a comprehensive and integrated analysis of the various legal dimensions applicable to the most basic kind of human genomic data—*personal genomic sequence data*, i.e., the genomic sequence data (often referred to as the “raw” genomic data) of a particular person (in contradistinction from aggregated genomic sequence data of multiple persons). We show that personal genomic sequence data exist in multiple legal dimensions simultaneously. We provide the reader with a comprehensive picture of the legal nature of personal genomic sequence data from the perspective of South African law, by analyzing how these dimensions interact with each other, and conclude that a bundle of rights is applicable to personal genomic sequence data. This conclusion, we argue, is significant because it illustrates how every dimension must be thoroughly understood before decisions are made regarding any of the individual rights (such as privacy rights), lest how that right is used or governed conflicts with other parties’ rights.

To best understand our main argument, consider this everyday example: If you take a photograph of something and are responsible for the composition of the photograph, you are automatically the owner of the copyright in the photograph—irrespective of the photograph’s artistic merit. You can assign the copyright or license use of it. This is different to owning a physical print of the photograph. You can make hundreds of prints of the photograph and sell these prints to various people without losing your copyright in the photograph. This demonstrates the difference between the intellectual property law dimension and the property law dimension. To demonstrate the personality rights dimension, consider the following expansion on the hypothetical facts: If the photograph you took was an intimate photograph of another person, and you sold the prints of said photograph without his or her consent, this would clearly be an infringement of that person’s personality rights, and that person would be able to take legal action against you. The fact that you own the copyright is no defense. Your *intellectual property rights* in the photograph are limited by the *personality rights* of the person that appears in the photograph. Similarly, the person whose personality rights have been infringed may even be able to claim the physical prints from their owners, meaning that personality rights may, in certain circumstances, outweigh property rights. The point is that it would be erroneous to think of photographs only in terms of intellectual property rights, property rights, or personality rights—*all* these dimensions are applicable and interact with each other. The same is true for personal genomic sequence data.

### 1.1 Roadmap

We first provide clarity regarding the concepts and terminology used in this article—most pertinently, differentiating personal genomic sequence data from DNA, and defining what is meant by legal terms such as “property” and “ownership” in South African law. Our analysis of the various legal dimensions of personal genomic sequence data is divided into three parts. We first investigate the property rights dimension by considering the question of whether personal genomic sequence data qualify as property. After answering this question in the affirmative, we consider who the owners of personal genomic sequence data are, and whether there are alternatives to private ownership of such data. We then turn our attention to the personality rights dimension, where we analyze how data subjects’ personality rights in personal genomic sequence data interact with property rights in such data. We finally focus on the intellectual property law dimension, where we consider how personal genomic sequence data are utilized in genomic datasets, patents, and trade secrets, and how these intellectual property rights interact with both property rights and personality rights in personal genomic sequence data. We also highlight legal considerations applicable to intellectual property that result from publicly funded research in South Africa. We conclude by considering the practical implications of the bundle of rights applicable to personal genomic sequence data.

### 1.2 Conceptual clarity

It is important to conceptually differentiate personal genomic sequence data from the object from which such data are derived—namely DNA (Thaldar, 2021). DNA encodes and contains genomic information in the form of the sequence of the nucleotides. However, in such a pre-sequencing state the genomic information is not yet in a usable format for scientific research. Only once genomic information is derived from the DNA through a sequencing process—and acquires a separate existence as data stored on a computer—does it become usable for scientific research. In this article, to reflect the post-sequencing status of genomic information as being collected for analysis and stored on computers, we refer to it as personal genomic sequence *data*.

Personal genomic sequence data, similar to other digital objects, such as software, digital money, and electricity, are *fungible*. This means that multiple copies—or “instances”—of the same personal genomic sequence data can be made, and also that research participants can provide their tissue for genomics research to any number of research institutions. This results in all such research institutions independently generating instances of the same data. While the fungible nature of personal genomic sequence data may seem complex, there are many everyday, age-old examples of fungible objects—such as printed books. This is why one can say: “the Bible states that…” (referring to the Bible in the abstract), and: “my Bible has a page missing” (referring to a specific copy of the Bible). The same applies to personal genomic sequence data—the context will determine whether one refers to personal genomic sequence data in the abstract (e.g., “This research participant’s personal genomic sequence data contains…”) or to a specific instance of personal genomic sequence data (e.g., “I have transferred the research participant’s personal genomic sequence data to the external hard disk”).

### 1.3 Note on legal terminology

For the purposes of this discussion, the term “property” denotes a legal object (or “thing”) that is susceptible of ownership. Property can be corporeal (such as a house) or incorporeal (such as intellectual property). Ownership is the most comprehensive right that a person can have in property, and includes, most pertinently, the rights to use, alienate, enjoy the fruits of, and dispose of the said property (Thaldar and Shozi, 2021). However, ownership is seldom unrestricted. The state and other individuals with rights in the property may circumscribe the way in which owners may exercise control over their property. For example, a car owner must adhere to the rules of the road when driving on a public road. It is also important to note that an owner need not always be in actual physical control of the property, and may allow others to use it without relinquishing his or her rights to the property.

The term “custodian” is often used in the literature on genomic data governance (see, e.g., ASSAf and DST, 2018; Ramsay, 2022; Verlinden et al., 2016; Shah et al., 2019; Murtagh et al., 2018; Marelli et al., 2021; Suver et al., 2020). Thus, it is important to consider what this term means in relation to property. In Roman law, *custodia* denoted a liability standard applicable across a number of nominate contracts relating to property. (Nominate contracts are classified as belonging to an established type of contract with certain *naturalia*—i.e., implicit legal consequences. For example, the contract of purchase and sale automatically entails a guarantee against hidden flaws. Parties are free to then venture beyond the scope of nominate contracts and design their own unique contracts—called innominate contracts—but in such cases they should take care to provide for all eventualities, as the automatic legal protections of nominate contracts will not apply.) *Custodia* (*qua* liability standard) entails that in the event of the loss of the owner’s property that was placed in the custodian’s care, the custodian would be strictly liable for such loss—irrespective of the custodian’s own fault (Swart v Shaw; BC Plant Hire CC v Grenco (SA) (Pty) Ltd). As such, using the term “custodian” in South African law is insufficient to indicate the exact type of nominate contract that is intended, and would likely be interpreted as demanding not only the general standard (BC Plant Hire CC v Grenco (SA) (Pty) Ltd) of reasonable care of the custodian, but also a higher standard of *meticulous* care (Mercurius Motors v Lopez).

Assuming, for the sake of argument, that research participants are the owners of their genomic data and that research institutions are the custodians of such data (which is a possible but improbable scenario, as we discuss below), we suggest that the most suitable nominate contract would be *commodatum* (loan for use), as this contract is gratuitous and would allow research institutions to use the genomic data, in contrast with a contract such as *depositum* (safekeeping), which would render any use of the genomic data by the research institution unlawful. Our point here is that words such as “custodian” and “safekeeping” have legal technical meanings that may trigger unwelcome legal consequences. Accordingly, when drafting any document that is intended to have legal effect in South Africa, professional legal advice should be obtained.

Our analysis focuses on the generation of personal genomic sequence data by research institutions—broadly defined as entities, whether public or private, that spend a significant portion of their resources on research. Our focus on research institutions is a pragmatic choice, given that in South Africa, in the context of genomics, the generation of personal genomic sequence data would typically be done by research institutions. There may, of course, also be cases where personal genomic sequence data are generated for diagnostic or heritage purposes. However, for purposes of this article, we limit our analysis to the research context.

## 2 Analysis of the various legal dimensions

We start our analysis with the most under-investigated legal dimension of personal genomic sequence data: its common law property rights dimension. This is followed by the personality rights dimension and the intellectual property law dimension. Throughout, we highlight how these dimensions interact with each other.

### 2.1 Personal genomic sequence data: The property rights dimension

#### 2.1.1 Are personal genomic sequence data property?

Over the ages, several incorporeal things have made their appearance in society and have subsequently been recognized as new species of property. For example, in the year 1770, an English court referred to stock in a company as follows: “this is a new species of property, arisen within the compass of a few years” (Nightingale v Devisme). In recent times, the South African courts have held that digital money (Nissan South Africa (Pty) Ltd v Marnitz) and electricity (S v Ndebele) qualify as things that can be stolen, which necessarily implies that such objects are susceptible of private ownership (i.e., property). Furthermore, the South African Competition Tribunal accepted that retailers’ electronic point-of-sale data can be bought and sold (Competition Commission v British American Tobacco South Africa (Pty) Ltd), which necessarily implies that such *data* are property. In another case, the court, in passing, referred to the “ownership” of a phone number (S v De Vries) as a type of data. Recent literature in South African property law also supports the position that data are property (Erlank, 2015; Njotini, 2017).

Against this background, we now consider personal genomic sequence data. In South Africa’s common law—Roman-Dutch law—the criteria for something to qualify as property are that it must: 1) be useful and valuable; 2) not merely be part of something else; 3) not be part of a human body; and 4) be capable of human control (Thaldar and Shozi, 2021). Analogous to an instance of a software program or a copy of a book being the unit of analysis in property law (rather than the program or book in the abstract), the most promising unit of analysis for personal genomic sequence data is an *instance* of personal genomic sequence data, i.e., a single copy of a person’s genomic sequence data stored on a single device. Accordingly, when we refer to personal genomic sequence data in the analysis below, we do not mean personal genomic sequence data in the abstract, but an instance of personal genomic sequence data. We now consider criteria 1) to 4):

(1) That personal genomic sequence data are useful and valuable (academically and commercially) is manifest.
(2) Personal genomic sequence data exist separately from the DNA from which such data were derived. Furthermore, although personal genomic sequence data are recorded on one or more electronic devices, personal genomic sequence data are not bound to any particular device and can be moved from one recording device to another. Personal genomic sequence data are not merely part of something else.
(3) Although personal genomic sequence data are derived from DNA, which in turn is derived from the human body, personal genomic sequence data are generated outside of the human body and exist outside of it.
(4) Humans can decide, *inter alia*, on which device personal genomic sequence data are recorded, who has access to the data, how the data are processed, and for what purpose the data are processed. Thus, personal genomic sequence data can indeed be controlled by humans.

Accordingly, personal genomic sequence data—understood not in the abstract, but as a specific instance of personal genomic sequence data—qualify as property.

#### 2.1.2 Who owns personal genomic sequence data?

The next step in our analysis is to investigate how someone acquires ownership in property—personal genomic sequence data in this case. Personal genomic sequence data are not generated out of nothing—it requires technical expertise, the necessary laboratory infrastructure, and human biological material from which DNA can be extracted. Various individuals and institutions (and indirectly the state and other funders) may therefore contribute elements to the creation of personal genomic sequence data. There may be a number of aspirant owners. Since the default position in South African law is contractual freedom—in the sense that persons are free to enter into contracts and that these contracts are legal and binding (Barkhuizen v Napier)—those who contribute to the creation of personal genomic sequence data can determine, through contract, who the owner of the personal genomic sequence data would be, and on what terms.

That said, this default position of contractual freedom is significantly constrained through statute law in the context of research. In particular, the South African National Health Act 61 of 2003 (NHA) provides, in section 60(4), that it is an offence for a person who has donated tissue to receive any form of financial *or other reward* for such donation, except for the reimbursement of reasonable costs incurred by him or her. Having one’s genomic data generated as a new legal object and becoming the owner thereof *may* be considered a reward. If so, it would be unlawful to enter into a contract with a research participant, where the contract provides that the research participant would be the owner of the personal genomic sequence data (that is to be generated using the DNA derived from the tissue that is to be donated by such a research participant). Accordingly, if becoming the owner of genomic data qualifies as a reward for the purposes of section 60(4), the only candidates for ownership determined through contract would be the research institution(s) involved, and if there are private or public funders—such funders. Whether becoming the owner of genomic data qualifies as a reward is an important question that we intend to investigate in subsequent articles.

What would the legal position be in the absence of a contract? Given that data—*qua* property—are a new phenomenon in our law, it is not certain which of the modes of acquisition of ownership that exist in South African law would apply. The most likely candidate would be *occupatio* (appropriation), which entails that a person acquires ownership in respect of an object that belongs to no one by *intending* to be its owner and taking physical *control* of it. As such, it would require a construction that after its creation, personal genomic sequence data are property that belongs to no one. A potential obstacle to conceiving of data in this way could be the requirement of *physical* control, given that personal genomic sequence data are not a corporeal object. It may be *recorded* on physical devices, but the data and the recording device are not to be confused—they are distinct objects. In any event, the data may be recorded in the cloud, meaning that the researchers who did the sequencing may not even know the physical location of the computer servers where the data are recorded—much less have *physical* control over such servers.

We suggest that the requirement of physical control is outdated in today’s world where so many valuable assets have a digital rather than a physical existence, and where these digital assets are *effectively* controlled *via* digital device interfaces. For example, effective control may be held in the form of access codes, encryption keys, or passwords (or a combination of such measures). South African common law is dynamic and has readily recognized such forms of control in other contexts—e.g., in the context of international trade by sea, a bill of lading is legally recognized as giving the holder control over the goods recorded therein (Sanders Bros v Maclean & Co). We thus suggest that the intention to be the owner, plus *effective* control of personal genomic sequence data as a digital object, should suffice to acquire ownership through appropriation. This clearly favors the research institution that conducts the sequencing, as it will be in effective control of the data. If a research institution outsources the sequencing to a service provider, and assuming that the institution intends to claim ownership of the data, the institution should ensure that its contract with its service provider contains carefully crafted ownership provisions.

A word of caution when considering who owns personal genomic sequence data. Certain scholars (see, e.g., Hand, 2018) make the mistake of thinking that if someone has certain rights in respect of personal genomic sequence data that are typically entailed by ownership, such as the right to use, that such a person is the owner. However, this is to confuse a consequence with a criterion. Having the right to use something is a typical consequence of acquiring ownership in it, but it is not always the case and is by no means a criterion for ownership. For example, a person may buy (and become the owner of) a beach house, but if there was another person leasing the house from the previous owner, such a tenant will have the right to use the beach house for the duration of the lease agreement. The fact that the tenant is using the beach house does not make him or her the owner thereof. Similarly, the fact that the South African Protection of Personal Information Act 4 of 2013 (POPIA) provides that a data subject, i.e., the person to whom the personal information relates, has certain rights in respect of his or her data, does not establish ownership. It does, however, carve away at ownership. We explore this in further detail below.

#### 2.1.3 Are there alternatives to private ownership of personal genomic sequence data?

Private ownership is not the only option for personal genomic sequence data. If personal genomic sequence data are released into the public domain (unencrypted and without a non-fungible token (NFT)), they would arguably become the common heritage of humankind, similar to the air around us. Another possibility is that the state can—through statutory intervention—make all personal genomic sequence data *public* property, similar to public roads and mineral resources. This would entail that the state hold personal genomic sequence data in trust for the benefit of its subjects and regulate its use accordingly. This possibility has featured in recent policy proposals (ASSAf and DST, 2018; Draft National Data and Cloud Policy GN 306 of GG 44389, 2021).

But what about empowering individuals? Through statutory intervention, the ownership of personal genomic sequence data can be made to initially vest in the individual data subject. This policy option appears better aligned with the recognition by South Africa’s Constitution of each individual as an autonomous moral agent. At a practical level, technology may offer tools for individual persons to manage their personal genomic data. For example, smartphone apps that use blockchain infrastructure can be used to enable individual data self-governance and incentive-based data sharing in a privacy-preserving environment (Jin et al., 2019; Shabani, 2019; Carlini et al., 2020). This would radically *democratize* control of personal genomic sequence data.

However, the purpose of this article is not to engage in a normative analysis (what should the law be?), but to engage in a positivist analysis (what is the law?) that draws attention to the multidimensional legal nature of personal genomic sequence data. Accordingly, we do not develop these normative arguments further in this article. Nevertheless, it is noteworthy that technological solutions are emerging that can make data self-governance and digital rights management a reality: blockchain holds enormous potential to advance genomics research (see, e.g., Alghazwi et al., 2021; Mackey et al., 2019; Ozercan et al., 2018; Roman-Belmonte et al., 2018; Shabani, 2019; Thiebes et al., 2020a; Thiebes et al., 2020b). An NFT is a blockchain ledger entry that records ownership of a digital asset or linked physical object. The essence of a NFT is that it is unique, unlike ether and ERC-20 tokens which are interchangeable (fungible) in a manner similar to traditional and crypto-coins. Recently, genomic marketplaces, such as Nebula Genomics and Genobank, began pioneering the use of NFTs to ensure full traceability and portability of genomic data stored on blockchain platforms (Pennic, 2021). The privacy concerns about genomic data are well documented (see, e.g., Han, 2018; Gitschier, 2009; Naveed et al., 2015; Rocher et al., 2019; Wang et al., 2017), and are receiving the attention of researchers looking into permissioned *versus* public blockchain platforms (Kuo et al., 2019), secure off-chain storage (Benisi et al., 2020), encryption (Bernabe et al., 2019), and self-sovereign identity (SSI)-based identity and access management controls (Belchior et al., 2020; Kang and Lemieux, 2021; Lemieux et al., 2021; Papadopoulos et al., 2021). Although the technology is in its infancy, creating an NFT to record an individual’s genomic data, in conjunction with self-executing smart contracts, could provide individuals with control over who has access to their data and what the data are used for (Uribe and Waters, 2020). While NFTs can be commercially traded, they can also simply serve the purpose of being a permanent, immutable, and private record of one’s genomic data, which may serve as a non-monetary incentive to research participants (Benisi et al., 2020). Interestingly, by using this new technology, persons may be able to, for all practical purposes, change the nature of their personal genomic sequence data from fungible to non-fungible!

### 2.3 Ownership transcended (and restricted): The personality rights dimension

Every person is the holder of common law *personality* rights. In South African law, these are rights that are inseparably bound to one’s personality, which cannot exist independently of the human personality, and are incapable of being transferred (Kumalo v Cycle Lab (Pty) Ltd). Examples are the right to the integrity of a person, to respect a person’s name, reputation, and—importantly in this context—a person’s right to privacy (O’Keefe v Argus Printing and Publishing (Pty) Ltd). But what does it mean to have a personality right to *informational* privacy? It means that a person has the right to decide when and under what conditions private facts may be disclosed, and the ambit of disclosure (National Media Ltd v Jooste). Such a right is an important part of safeguarding a person’s broader privacy interests and facilitates free and just social interaction (Townsend, 2022). It is a right that a person has against another (the acquirer, holder, and controller of the data) and one that vests in the data subject by virtue of the sensitive and personal nature of, in this case, the personal genomic sequence data.

As we have demonstrated, data can be privately owned. While ownership of the data grants the owner the authority to determine how the object is used, in the case of *personal* data this right to use the data is restricted by the personality right of the data subject. A relationship thus exists between the data subject *qua* personality right holder and all third parties, including the owner of the data and all subsequent owners, who must respect the entitlements of the data subject. The personality right exists concurrently with (and independently of) the right of ownership, just as a copyright in the dataset may exist independently of the other rights and be held by a separate right holder. The personality right (to informational privacy) is thus a separate right which arises on the creation and recording of the data, and exists independently of the property right.

The United Nations Educational, Scientific and Cultural Organization (UNESCO) International Declaration on Human Genetic Data (2003) contains provisions that relate to personality rights in respect of “human genetic data”, which it defines as “Information about heritable characteristics of individuals obtained by analysis of nucleic acids or by other scientific analysis”. This would therefore include personal genomic sequence data. Although the Declaration is not legally binding, South African courts can consider the Declaration when developing personality rights in respect of human genetic data. In brief, the Declaration provides that individuals must give consent “without inducement by financial or other personal gain” for the collection, storage, and processing of their genetic data, and further that individuals must have the option to withdraw consent, unless the relevant data are “irretrievably unlinked” from an identifiable person. The Declaration places a duty on the state to protect individual privacy and safeguard the confidentiality of identifiable data.

In South Africa, much of the substance of the common law personality right in respect of personal data has been codified (i.e., made into statute law) and expanded on in POPIA. The protections offered by POPIA prevent and limit the unlawful processing of personal information by stipulating when, how, for what purpose, and by whom a data subject’s personal information may be collected, used, or shared. In terms of POPIA, for such protection to be exercised, the data must first be “personal” information as defined, that is, information relating to a person, including, but not limited to, the biometric information of the person. Personal genomic sequence data would fall within this definition. Secondly, it must have been recorded or reduced to a “record”, regardless of when it came into existence. A “record” is defined in POPIA as any recorded information, regardless of form or medium, produced, recorded, or stored by means of any computer equipment, including hardware, software, or other device, and any material subsequently derived from such information. As whole-genome sequencing using high-throughput sequencing technology is not an instantaneous event, but rather a gradual digital accumulation of genetic information over a period of hours, the recording of personal genomic sequence data in digital form is also not an instantaneous event, but rather a process (Thaldar, 2021). Lastly, for POPIA to apply, the data must not have been de-identified. De-identification is the process used to prevent revealing the identity of the person to whom the data relates. Whether *genomic data* can ever be truly de-identified is the subject of much recent conjecture (Shabani and Marelli, 2019; Townsend and Thaldar, 2019). Rocher et al. (2019) suggest that even heavily sampled anonymized datasets are unlikely to satisfy the modern standards for anonymization, and challenge the notion that technical and legal de-identification is possible. Although personal genomic sequence data are uniquely identifiable of an individual, they cannot be used to identify that individual unless there is a link to personally identifiable data. Importantly, the mere fact that personal genomic sequence data—or any genomic data—have been separated from any identifying information about the person to whom it relates, does not mean that such data are legally de-identified. If there is a *reasonably foreseeable method* to link such data, the personal genomic sequence data would fall foul of the definition of “de-identify” in POPIA.

As a personality right is inalienable and cannot be lost or separated from the data subject’s personal data, the data subject (*qua* personality right holder) can exercise the independent entitlements connected to the personality right, as well as those provided for in POPIA. Such an entitlement grants data subjects control, access, and use of their personal data. In the research context, the ways in which personal data can be used by a third party (in this case, the research institution) can then be curtailed at the data subject’s discretion through the right to withdraw consent, to be notified of, and to object to the processing of their personal data. So, although the third party may *own* the data, in effect the owner’s entitlements are curtailed in a specific way: they do not have unfettered leeway to collect, use, and share the personal genomic data, and any subsequent use and research must be subject to such personality rights of the data subject (and as contained in POPIA). Aligned with this, POPIA provides for the future use and re-purposing of personal data under certain circumstances. In this regard, the further processing of personal information for research purposes is allowed—subject to the condition that the data must not be published in an identifiable form. We caution that although this “research exception” may permit secondary analysis of data for further research, it does not exonerate researchers from complying with POPIA.

It is worth reiterating that the personality rights of research participants (data subjects) cannot be divorced from their personal data. Thus, ownership in the personal data exists in addition to, and independently from, and in a sense is encumbered by the independent entitlements afforded by the personality rights of the data subject. Any onward transfer of data ownership would, therefore, be burdened by the personality rights of the data subjects—including their POPIA rights—existing at the time of the transfer. For example, if a research institution were to transfer a dataset containing the personal genomic sequence data of several individuals to another research institution, the second instances of the personal genomic sequence data of those individuals (*qua* new legal objects) would be susceptible of ownership by the recipient research institution (if that was what was intended by the parties to the data-sharing agreement). However, the data would remain encumbered by the independent entitlements afforded by the personality rights of the data subjects (including POPIA) and the dataset as a whole would remain protected by copyright of the transferring institution.

Lastly, given the nature of personal genomic sequence data, which contains information not only about the data subject but also about the data subject’s ancestors, descendants, family, and ethnic group more broadly, the question can be posed whether such persons (or community or state) should not also have a personality or personality-type right in respect of such data. For present purposes, suffice to say that POPIA only protects the individual data subject. However, this does not bar development of the common law on personality rights. As such, this is rich soil for further research.

### 2.4 From data to datasets and beyond: The intellectual property rights dimension

Intellectual property refers to creations of the mind—such as inventions; literary, artistic, and musical works; designs; and symbols, names, and images used in commerce (WIPO, 2022). Personal genomic sequence data *per se*—also referred to as “raw” data—are not creations of the mind, and therefore do not attract intellectual property rights. However, personal genomic sequence data can be useful building blocks to create intellectual property. We consider three ways in which personal genomic sequence data are used: to compile genomic datasets, to develop new technologies, and as trade secrets.

#### 2.4.1 Datasets

In South Africa, if a person creates a compilation of personal genomic sequence data, i.e., a genomic dataset, *copyright* in such a dataset will automatically vest in such a person—as per the Copyright Act 98 of 1978 (Copyright Act). Importantly, copyright vests in the dataset as a whole, and not in the constituent personal genomic sequence data.

This raises the question of what the effect would be, in *property law*, of including an instance of personal genomic sequence data in a dataset. In principle, it would be possible to sell or rent out the use of the dataset, in addition to assigning or licensing out the copyright in the dataset. But does this mean that the personal genomic sequence data lose their status as legal objects and become mere parts of the dataset? We suggest not. This would only be the case if personal genomic sequence data become permanently part of a dataset; becoming part of the legal object that is the dataset by way of accession and thereby losing its status as an individual legal object. However, this is not so. The data of a particular constituent personal genome can, with relative ease, be separated from the dataset. Accordingly, it does not accede to the dataset. A useful comparison to illustrate this would be a flock of sheep. While the flock can be dealt with as a single economic unit, each sheep retains its individual legal object status, and can always be separated from the flock and be dealt with *qua* individual legal object. The same applies to a genomic dataset: while it is convenient to deal with it as a single economic unit that consists of multiple individuals’ personal genomic sequence data, the data of each constituent personal genome retains its status *qua* legal object.

This means that if compilers of genomic datasets do not acquire ownership of all the constituent personal genomic sequence data, they need to obtain the consent of all the owners of the constituent personal genomic sequence data to be able to out-license use of the dataset (or perform any other act that would affect the ownership rights of the owners of the constituent personal genomic sequence data). It would clearly simplify matters if a dataset were created by the same research institution that owns the constituent personal genomic sequence data. However, if multiple research institutions are involved in the provision of the constituent personal genomic sequence data and the creation of the dataset, a data management plan should be put in place to provide for issues such as ownership of the constituent data and how the dataset is to be used.

It is also important to note that the creation of a dataset of personal genomic sequence data and the consequent vesting of copyright in it, also does not diminish the privacy rights of the data subjects whose genomic sequence data comprise the dataset. If using the dataset falls within one of POPIA’s research exceptions, consent from the data subject need not be obtained to use (or out-license use of) the dataset. However, if use of the dataset is to be out-licensed to someone beyond the borders of South Africa, POPIA sets additional requirements that need to be complied with (Townsend, 2022).

#### 2.4.2 Patents

In the present context, a patentable invention may be in the form of a diagnostic tool, a therapeutic composition, or, since South African courts are likely to take into account Article 5 of the European Biotechnology Directive (Directive 98/44/EC of the European Parliament and of the Council on the legal protection of biotechnological inventions, 1998), one or more isolated gene sequences—provided they have a demonstrated functional use. Acquiring a patent on an invention requires that an application be filed with a patent office, which is then examined and, if granted, is published in a patent register. This is in contrast with intellectual property in the form of proprietary confidential information (discussed below), where industry know-how is not published, but kept secret. Patents require disclosure in the published patent specification on how to make and use a particular invention. Patents originated as a state measure to promote industrial innovation through widespread dissemination of knowledge, while at the same time incentivizing innovation. This is achieved by providing inventors with temporary exclusive commercial rights in respect of their inventions, provided that the invention is novel, involves an inventive step, and is capable of being used or applied in trade, industry, or agriculture.

Importantly, in order for an inventor to patent an invention, the inventor does not need to own the object that the invention relates to. Applied to the present context, an inventor does not need to own a personal genomic sequence data fragment—a gene sequence—in order to patent such a gene sequence. Similarly, the fact that the object that the invention relates to is also the object of privacy rights does not necessarily impede the invention’s patentability. One should remember that patents do not bestow a right to use the invention. In order to use a patented gene sequence, permission would be required from both the owner of such data and the data subject.

In this context, it is interesting to note that the South African Patents Act 57 of 1978 (Patents Act) provides that a patent may not be granted for an invention where its publication or exploitation would generally be expected to encourage offensive or immoral behavior. Provisions such as these exist in patent law to ensure that patents are not used in a manner that is contrary to the public interest. However, this provision in the Patents Act has never been tested in the courts. We suggest that the fact that third parties have property or privacy rights in relation to the object of a patent application is not *per se* offensive or immoral. What would an offensive or immoral scenario look like in the genomics context? Consider the following hypothetical scenario: An inventor applies for a patent on a diagnostic method, or a method of selecting a medical compound or composition for use in the treatment of a particular individual or ethnic group. The invention relates to a gene sequence obtained from an individual or persons belonging to such an ethnic group. However, in a wicked turn of events, the inventor intends to commercialize the patent in such a way as to make it unreasonably expensive to access, or the inventor intends not to commercialize the patent at all and only use it to block potential competitors. In this scenario, we suggest that the Patents Act’s offensive or immoral provision would be triggered. Ergo, patent rights relating to genomic data should be used in a manner that is not only respectful of individuals’ privacy rights and ownership rights, but also for purposes that are aligned with the public interest.

#### 2.4.3 Trade secrets

In South Africa the protection of proprietary confidential information, including trade secrets, is not legislated by statute, such as in the case of patentable inventions, but rather its protection relies on the common law. The misappropriation or unauthorized use of proprietary confidential information is considered to be *prima facie* wrongful, and is actionable under the law of delict, the law of unlawful competition, or the law of contract (where a party is in breach of a contractual obligation of confidentiality) (Meter Systems Holdings Ltd v Venter; Knox D’Arcy Ltd v Jamieson; Waste Products Utilisation (Pty) Ltd v Wilkes; Hirt Carter (Pty) Ltd v Mansfield).

In keeping with the Agreement on Trade-Related Aspects of Intellectual Property Rights (1994) (TRIPS Agreement), the common law provides the general requirements for confidential information to qualify as a trade secret, i.e., that such information must: 1) be capable of use in trade or industry; 2) have economic value to the proprietor; 3) not be public knowledge; and 4) be maintained by the proprietor (trade secrets are not owned, but possessed) as a secret. Economic value is typically evidenced by the potential or actual usefulness that the confidential information would have should a rival gain access to it, and the amount of work, skill and time required to produce the trade secret. We suggest that if requirements 1) to 4) are met with regard to personal genomic sequence data, such data would qualify as trade secrets. Accordingly, assuming that owners of personal genomic sequence data are also in possession of such data, if they keep the data secret (for example, by using non-disclosure agreements with employees and research partners), they will enjoy trade secret protection against misappropriation or unauthorized use.

Given the fungible nature of personal genomic sequence data, the question arises as to whether an individual’s personal genomic sequence data can ever be maintained as secret by a research institution, as the data subject can conceivably provide biological samples to any number of other research institutes for genomic sequencing. Data subjects can even, in theory, decide to publish their genomic data online—open for anyone to use. We suggest that these possible eventualities are of a practical nature and do not, in principle, disqualify personal genomic sequence data as trade secrets. The requirement is that the relevant personal genomic sequence data must, as a matter of fact, not be public knowledge. However, these possible eventualities illustrate that trade secret protection does not limit the rights of the data subject—instead, it is *vice versa*: the rights of the data subject can destroy a trade secret. Accordingly, where a research institution is the owner of personal genomic sequence data, trade secret protection can add a useful weapon to its arsenal of legal remedies that can be used *vis-à-vis* other research institutions, but not against the data subject. Note that the benefit of trade secret protection comes at the cost of a self-imposed restriction on ownership, namely keeping the personal genomic sequence data confidential.

As with patentable inventions, it is important to remember that the proprietor’s right to use or commercialize personal genomic sequence data is subject to the data subject’s privacy rights, as well as statutory limitations on use and commercialization, such as those provided in the NHA.

#### 2.4.4 Intellectual property from publicly funded research

No discussion of intellectual property rights in South Africa is complete without mentioning that intellectual property that emanates from research that received financial support from the South African government—no matter how small—is subject to an additional layer of regulation, in terms of the South African Intellectual Property Rights from Publicly Financed Research and Development Act 51 of 2008. The National Intellectual Property Management Office (NIPMO) exercises a wide range of statutory powers, and is mandated to ensure that such intellectual property is “protected, utilized and commercialized for the benefit of the people of the Republic” (Intellectual Property Rights from Publicly Financed Research and Development Act 51 of 2008). While this mandate restricts the rights of the owners of affected intellectual property rights, it does not restrict the ownership rights of personal genomic sequence data owners or data subjects whose personal genomic sequence data are contained in genomic datasets.

## 3 Conclusion

The best metaphor to describe the multidimensional legal nature of personal genomic sequence data is a *bundle of rights* applicable to personal genomic sequence data. This bundle of rights may include common law ownership, personality rights, and intellectual property rights. The interplay between these various rights will determine the measure of control over the personal genomic sequence data that different stakeholders, such as research institutions, research participants, research funders, and any downstream entities that wish to commercialize innovations derived from or encompassing personal genomic sequence data, can lawfully exercise at various steps in the innovation process.

The idea that personal genomic sequence data are susceptible of ownership may be new and still under-investigated, but, as we have shown in this article, if the well-established common law criteria are applied, personal genomic sequence data qualify as being susceptible of private ownership. We however need to emphasize that our analysis in this article is of a legal positivist nature, and not of a normative nature. In other words, we investigated what the law is, and not what the law ought to be. This distinction is important, as legal reality cannot be changed by subjectively willing something different. For example, avoiding the *language* of ownership in personal genomic sequence data in policy discourse or genomic research-related documents will not change the reality that it is a legal fact.

The practical consequence of the bundle of rights applicable to personal genomic sequence data is that public policies and institutional policies should acknowledge and deal with personal genomic sequence data in all of these dimensions. In other words, a policy that deals with personal genomic sequence data only in one or two dimensions leaves a lacuna that can lead to legal uncertainty or undesirable consequences. For example, consider a scenario where a research institution collects biological samples from human research participants, extracts the DNA, and sequences the genomes. However, the research institution has no agreements in place with the research participants regarding ownership of the personal genomic sequence data. Furthermore, the research institution labors under the misapprehension that personal genomic sequence data are not susceptible of ownership, and therefore never forms the intent to acquire ownership. The result is that personal genomic sequence data are generated, but nobody acquires ownership in such data—at least not at the time that the data are generated. Personal genomic sequence data are *res nullius*—something that belongs to no one. Why is this problematic? Because the first person who intends to become the owner of a *res nullius* and takes control of it, acquires ownership in it. To expand on the scenario described above: What if the research institution shares the personal genomic sequence data with its international consortium partners, but still fails to address ownership of the data in the consortium agreement? At that stage, any one of the consortium partners can lawfully acquire ownership in the data—at least their copy of it—simply by willing it and being in effective control of their copy of the data. Alternatively, the research institution compiles a dataset and out-licenses use of the dataset to a foreign company. Again, if the license agreement does not prohibit the licensee from claiming common law ownership in the data in the dataset, the licensee can easily and lawfully acquire ownership in the data. This may have drastic, unintended consequences for the research institution that initially generated the data, but which neglected to properly deal with the ownership dimension of personal genomic sequence data. This underscores the importance of dealing with all of the legal dimensions of personal genomic sequence data—as notably highlighted in this article.

## Data Availability

The original contributions presented in the study are included in the article/supplementary material, further inquiries can be directed to the corresponding author.
